# Perceptual and Social Attributes Underlining Age-Related Preferences for Faces

**DOI:** 10.3389/fnhum.2016.00437

**Published:** 2016-08-31

**Authors:** Hanni S. M. Kiiski, Brendan Cullen, Sarah L. Clavin, Fiona N. Newell

**Affiliations:** Multisensory Cognition Research Group, Institute of Neuroscience and School of Psychology, Trinity College DublinDublin, Ireland

**Keywords:** face perception, aesthetic preferences, aging, attractiveness, character traits

## Abstract

Although aesthetic preferences are known to be important in person perception and can play a significant role in everyday social decisions, the effect of the age of the observer on aesthetic preferences for faces of different ages has not yet been fully investigated. In the present study we investigated whether aesthetic preferences change with aging, with an age-related bias in favoring faces from one’s own age group. In addition, we examined the role of age on both the perceptual qualities and the social attributes of faces that may influence these aesthetic judgements. Both younger and older adult observers provided ratings to images of younger, middle-aged and older unfamiliar faces. As well as attractiveness, the rating dimensions included other perceptual (distinctiveness, familiarity) and social (competence, trustworthiness and dominance) factors. The results suggested a consistent aesthetic preference for youthful faces across all ages of the observers but, surprisingly, no evidence for an age-related bias in attractiveness ratings. Older adults tended to provide higher ratings of attractiveness, competence and trustworthiness to the unfamiliar faces, consistent with the positivity effect previously reported. We also tested whether perceptual factors such as face familiarity or distinctiveness affected aesthetic ratings. Only ratings of familiarity, but not distinctiveness, were positively associated with the attractiveness of the faces. Moreover, ratings of familiarity decreased with increasing age of the face. With regard to the social characteristics of the faces, we found that the age of the face negatively correlated with ratings of trustworthiness provided by all observers, but with the competence ratings of older observers only. Interestingly, older adults provided higher ratings of perceived competence and trustworthiness to younger than older faces. However, our results also suggest that higher attractiveness ratings, together with older aged faces, led to more positive evaluations of competence. The results are discussed within the context of an age-related decline in the differentiation of faces in memory. Our findings have important implications for a better understanding of age-related perceptual factors and cognitive determinants of social interactions with unfamiliar others across the adult lifespan.

## Introduction

Our aesthetic preferences for other people govern many aspects of our life; they influence our choices from romantic partners to political leaders (Langlois et al., 2000; Cornwell et al., 2006; Olivola et al., 2014), sometimes even when more objective information is available (Todorov et al., 2015). Moreover, these aesthetic preferences are often influenced by the physical features of a face (e.g., Valentine et al., 2004), and can be rapidly determined from a glance at a face of a person we may never have encountered before (i.e., the ‘zero acquitance effect’; Albright et al., 1988). Such rapid perception suggests the importance these impressions have for decision-making in everyday, social contexts.

Although the conventional wisdom is that ‘beauty is in the eye of the beholder,’ a large body of literature exists to contradict this claim by showing that perceived attractiveness is highly consistent across raters of different cultural backgrounds or influences (e.g., Langlois et al., 2000; Apicella et al., 2007). To date, however, most studies on facial attractiveness have drawn their findings from younger adult populations evaluating young adult faces, and the importance of age on attractiveness judgments has not yet been extensively investigated. As a result, much of what we understand about aesthetic preferences for faces may be skewed by this sample. This previous emphasis on youth is surprising given the increasingly aging population in Western societies. Furthermore, the growing number of studies from social psychology suggesting that older adults are particularly affected by fraud and deception (Ruffman et al., 2012), thought to be specifically linked to face perception (e.g., Ruffman et al., 2006), suggests a great need for understanding age-related factors that affect person perception and the determinants of preferences for faces as we age. Such knowledge can, in turn, provide a better insight into the perceptual basis of social outcomes in everyday life situations (Langlois et al., 2000; Cornwell et al., 2006; Olivola et al., 2014).

Although all faces share the same basic configuration of features, we are sensitive to the subtle “surface” features and their age-associated changes when determining the age of a face (e.g., Burt and Perrett, 1995). Age-related changes in the shape of the face as well as skin texture, and colouration, are important for evaluations of attractiveness and health (Burt and Perrett, 1995; Bruce and Young, 1998). However, besides the age of a face, the age of the observer may also affect judgements of facial attractiveness. For example, previous research in face perception suggests evidence for an own-age bias (Wright and Stroud, 2002; Anastasi and Rhodes, 2005; Rhodes and Anastasi, 2012) with better recognition and identification of previously unfamiliar face stimuli that match one’s own age group. Also, both older and younger adults have been reported to show accentuated facial stereotypes for faces similar to their own age (Zebrowitz and Franklin, 2014). These effects may be driven by a perceptual benefit for processing information from faces matching the observer’s own age group (Wiese et al., 2013). Finally, there is evidence for a positivity bias, in that older adults tend to give higher ratings to faces compared to young adults, and this effect is thought to be related to a decline in the ability to perceive differences in faces with increasing age of the observer (Mather and Carstensen, 2005; Ebner, 2008; Zebrowitz et al., 2013). For example, Ng et al. (2014) reported that older adults show less differentiation between faces in their ratings and concluded that this lack of making fine distinctions between face stimuli is reflected in older adults’ more positive impressions.

Previous attempts have been made to account for the perceptual factors underpinning the attractiveness of faces. For example, according to the ‘face-space’ model (e.g., Valentine, 1991; Valentine et al., 2015), the representation of a face in memory is influenced by properties such as its distinctiveness and familiarity relative to other faces. Furthermore, these two perceptual attributes of a face may also influence its perceived attractiveness. For example, increasing familiarity has been shown to enhance the positive affect of faces, known as the mere exposure effect (e.g., Peskin and Newell, 2004). Familiar faces are also liked more, and are judged to be more trustworthy, than unfamiliar faces (Zebrowitz et al., 2007; Sofer et al., 2015). Besides familiarity, the distinctiveness of a face has been indicated to be important both for the perception of unfamiliar faces (Light et al., 1979; Newell et al., 1999) and face memory (Courtois and Mueller, 1981; Shapiro and Penrod, 1986; Brigham, 1990; Sarno and Alley, 1997). In general, facial distinctiveness is associated with superior memory of that face, which may be due to enhanced encoding or retrieval of the unusual features of the face (Fleishman et al., 1976; Shapiro and Penrod, 1986; Newell et al., 1999). The attractiveness of a face has been found to be negatively associated with its distinctiveness (Rhodes and Tremewan, 1996), where increasing ratings of distinctiveness are associated with lower ratings of attractiveness. However, research on the effects of age on the perceived atttractiveness of a face has not hitherto taken into account the perceptual qualities of a face (e.g., Zebrowitz et al., 2013), such as its familiarity and distinctiveness, that can be affected by the aging process (e.g., Oosterhof and Todorov, 2008).

Apart from the perceptual correlates of faces underpinning aesthetic preferences, attractiveness ratings are often correlated with ratings along other dimensions relating to the social attributes of a face (Todorov et al., 2015). These important dimensions can be summarized as youthful-attractiveness, trustworthiness and dominance (Oosterhof and Todorov, 2008; Sutherland et al., 2013). For example, youthful-attractiveness conveys the reproductive quality of the face, whereas trustworthiness signals the perceived intention to help or harm and dominance reflects physical strength and the ability to perform these pro-social or anti-social intentions. Other studies have also reported other dimensions, such as ‘warmth’ (i.e., cooperation versus competition, that is arguably similar to trustworthiness) and ‘competence’ (i.e., status), which have been shown to be important for social evaluations across different types of stimuli, or across cultures and time (Fiske et al., 2007).

Previous studies have provided evidence for multiple processes influencing judgements of attractiveness. Importantly, the findings from neuroimaging and behavioral studies suggest a dissociation between processes underpinning aesthetic evaluations and sexual reward (Aharon et al., 2001; Franklin and Adams, 2009, 2010). This dissociation may be particularly pertinent with changes in either the age of the face or age of the observer. For example, Sutherland et al. (2013) proposed that the perceived age of a face was an important cue for deriving aesthetic evaluations. This is likely particularly true for youthful-attractiveness in female faces in particular, which signals reproductive value. However, it is possible that older and younger adults may differ in their motivations when evaluating the attractiveness of other faces. For example, mate quality may be an important determining factor in aesthetic evaluations of young faces but it is not clear whether this factor may also influence the evaluation of older faces. In particular, since fertility declines with age, such a factor may influence the aesthetic evaluations in an age-specific manner. However, the influence of observer age in determining the perceived trustworthiness or dominance of an unfamiliar face, across different ages of the face, has not been investigated thoroughly.

In recent years, data-driven approaches have identified the most crucial dimensions that sufficiently describe the numerous social evaluations derived from unfamiliar faces. A growing body of evidence suggests that forming an impression of a face is based not only on its attractiveness but also other social attributes, and is consistently found across all cultures (Fiske et al., 2007). It is argued that such impressions of the social attributes of a face can be rapidly achieved, i.e., within 34–100 ms (Willis and Todorov, 2006; Todorov et al., 2015). Furthermore, there is evidence for an ‘attractiveness halo effect’ in which faces perceived to be attractive are also rated more positively in other social attributes, such as competency (Dion et al., 1972; Langlois et al., 2000). Such correlations have also been shown to occur in ratings of older adults (Zebrowitz and Franklin, 2014).

The main objective of this study was to understand how aesthetic preferences for unfamiliar faces change across different ages of faces, and to investigate the importance of the age of the observer on these attractiveness judgments. We were also interested in whether there was a bias in a preference for faces from one’s own age group (i.e., an own-age bias). Our second objective was to investigate how perceptual dimensions (i.e., face qualities important to perceptual processing) of familiarity and distinctiveness, together with age, affect aesthetic preferences. We were also interested in elucidating the effect of age when determining the social attributes of a face (i.e., related descriptive qualities of a person), including competence, trustworthiness and dominance, and whether there was evidence for an attractiveness halo effect with increasing age. Furthermore, we investigated evidence for the positivity bias typically shown by older raters, and adopted the probability of differentiation approach to examine the level of distinctions made among stimuli by younger and older adults (i.e., whether older adults perceive faces less different from one another, see Ng et al., 2014).

In sum, based on previous findings from the literatures on the perception of faces and evaluation of social traits (e.g., Albright et al., 1988; Rhodes and Tremewan, 1996; Lee, 2001; Ebner, 2008; Zebrowitz et al., 2013; Ng et al., 2014; Zebrowitz and Franklin, 2014; Todorov et al., 2015) our main hypothesis was that the age of a face and age of the observer would each influence the aesthetic evaluations of a face. Specifically, we were interested in determining whether: (1) the ratings of attractiveness would decrease with the increasing age of unfamiliar faces but would increase from younger to older observers (due to the positivity bias); (2) the aesthetic ratings of a face would be modulated by an own-age bias; (3) that the perceptual dimensions of familiarity and distinctiveness, together with age-related factors, would influence aesthetic preferences from faces; (4) that attractiveness would have a halo effect on the evaluations of the social attributes of a face, which would also be modulated by the age of a face; and (5) that any differences in ratings between older and younger adults might be explained by a lower probability of differentiation in older relative to younger adult observers.

## Materials and Methods

### Participants

Sixty-five younger adults (21 males, *M*_age_ = 22.8 years, range = 18-33 years) and 46 older adults (17 males, *M*_age_ = 70.9 years, range = 57–87 years) participated in the experiment. All participants reported normal or corrected-to-normal vision and normal hearing, and were healthy at the time of testing. All young adults were students or staff in Trinity College Dublin and received either a small monetary reward (€5) or course credit to partake in the study. Older adults were recruited from the community and, for their participation, were compensated for any expenses incurred. Ethical approval was granted by the School of Psychology Research Ethics Committee, Trinity College Dublin. Accordingly, written informed consent was obtained from all participants in accordance with the Declaration of Helsinki before the onset of the experiment.

### Stimuli and Apparatus

All 162 face images (81 males) were acquired and used with a permission from the Max Planck Institute for Human Development Berlin FACES data base (Ebner et al., 2010). These 162 face images included faces of younger (27 males/females, *M*_age_ = 24.3 years, *SD* = 3.5, range = 19–31 years), middle-aged (27 males/females, *M*_age_ = 49.0 years, *SD* = 3.9, range = 39–55 years) and older (27 males/females, *M*_age_ = 73.2 years, *SD* = 2.8, range = 69–80 years) adults. As described in Ebner et al. (2010), all face images were identically lit, the images of the faces were a uniform distance from the edge of the picture and all images were of faces with a neutral expression. No further alterations were made to the original images of the faces.

The experiment took place in a quiet room and the participants were seated in front of a computer screen at a distance of approximately 60 cm. The experiment was programmed using Presentation^®^ software. The face stimuli presented on screen were 480 × 600 pixels and subtended visual angles of approximately 11.48° horizontally and 15.06° vertically.

### Design

The experiment was based on a mixed-design with the age of the face stimuli (younger, middle-aged, older) as a within-subjects factor, and the age of the participants (younger or older) and rating dimensions (i.e., attractiveness, competence, trustworthiness, dominance, familiarity and distinctiveness) as between-subjects factors. The rating score was the dependent variable.

### Procedure

The experiment consisted of two sessions, and each session required the participant to rate the faces on one trait dimension only. Each participant was given two trait questions. In total six traits were used for the study, i.e., attractiveness, competence, trustworthiness, dominance, familiarity and distinctiveness. All possible combinations were used and the order of the trait questions were counterbalanced across sessions for the younger and older participant groups separately. In turn, each session included six separate testing blocks of trials. Each block corresponded to one sex and age group of the face images, e.g., middle-aged male faces. These blocks were presented in a randomized order across participants. Following the completion of the first session of the experiment, all six blocks were shown again in the second session; however, the rating task was changed to reflect a different trait.

The participants were instructed to rate each face on a scale from 1 to 7, in which, for example, ‘1’ means ‘not very attractive’ whereas ‘7’ means ‘very attractive.’ Participants were asked to rate each face in comparison to the other faces in their age and sex group to ensure that variations in ratings would less likely reflect age or sex stereotypes. The questions and response options for each trait dimension used during the experiment are provided in **Table 1**. The participants were given instructions, first verbally and then were required to read the same instructions presented on the computer screen, before starting the experiment. After the presentation of each face image, the participant was prompted with a rating scale on the computer screen and asked to provide a response by using a standard PC keyboard to input the corresponding number on the rating scale. Participants were encouraged to consider the whole scale when making their response. Faces were shown for 1 s and immediately after the participant was presented with a trait question. Once a response had been made, the next trial was initiated. There was no time constraint for making a response although participants were encouraged to respond as soon as possible. Participants were offered self-timed breaks after each block and they completed the experiment within approximately 15–30 min.

**Table 1:** The questions, descriptions and response options for each trait dimension used during the experiment.

DIM	Question	Response options	
		1	2
ATT	How attractive is this person?	Not very attractive	Very attractive
FAM	How familiar is this person?	Not very familiar	Very familiar
DIST	How distinctive is this person?	Not very distinctive	Very distinctive
COM	How competent is this person?	Not very competent	Very competent
DOM	How dominant is this person?	Not very dominant	Very dominant
TRUST	How trustworthy is this person?	Not very trustworthy	Very trustworthy

*DIM, rating dimension; ATT, attractiveness rating dimension; FAM, familiarity rating dimension; DIST, distinctiveness rating dimension; COM, competence rating dimension; DOM, dominance rating dimension; TRUST, trustworthiness rating dimension.*

### Differentiation Scores

To complement the analyses of the data, we decided to also adopt a differentiation score approach. This approach allows us to calculate the probability that a rater will assign two randomly chosen faces to different levels on a rating scale, which is an indication of the level of age-related neural differentiation of the face stimuli (i.e., the cognitive processes become less distinct in older adulthood; e.g., Baltes et al., 1980; Salthouse et al., 1996; Lee et al., 2014). The differentiation scores for each rating dimension were calculated using the probability of differentiation (*P*_D_), where *i* is the level on the rating scale (1–7), and *P* is the proportion of ratings at the *n*th level (for more details see Linville et al., 1986; Ng et al., 2014). Thus, a high differentiation score (i.e., close to 1) indicates that the rater used more levels on the rating scale of a dimension when rating a series of faces on a particular dimension. The differentiation scores of each rater were computed separately for the sex and age groups of the face images (e.g., older males). We did this in order to control for the effect that a large variability in face images would have on the differentiation scores, i.e., greater differentiation scores would be expected for a set of face images that varied in sex and/or age.

## Results

### Older and Younger Adults’ Ratings of Attractiveness in Younger, Middle-Aged and Older Faces

The ratings of one older participant were excluded from all analyses due to not understanding the task (e.g., most of their responses were restricted to one key press only).

We calculated the extent to which participants agreed on their ratings of attractiveness using intra-class correlations (ICC). The inter-rater reliability score was high (*α* > 0.85) in both younger and older participant groups (see **Table 2**).

**Table 2:** The intraclass correlation coefficients (95% CI) and median ratings (95% CI) of younger and older participants for attractiveness, and the perceptual and social attributes of attractiveness.

		ALL	Y	M	O
**ATT**					
YA	ICC (95% CI)	0.92 (0.90,0.94)	0.88 (0.84,0.93)	0.88 (0.83,0.92)	0.89 (0.84,0.93)
	Median (95% CI)	2.63 (2.66,2.93)	3.46 (3.31,3.70)	2.50 (2.45,2.82)	2.13 (2.07,2.41)
OA	ICC (95% CI)	0.89 (0.86,0.91)	0.80 (0.71,0.87)	0.88 (0.83,0.93)	0.80 (0.71,0.87)
	Median (95% CI)	3.53 (3.40,3.68)	4.26 (4.07,4.41)	3.29 (3.23,3.68)	2.88 (2.75,3.09)
**FAM**					
YA	ICC (95% CI)	0.69 (0.61,0.75)	0.59 (0.42,.74)	0.64 (0.48,0.76)	0.74 (0.62,0.83)
	Median (95% CI)	3.21 (3.14,3.33)	3.32 (3.32,3.62)	3.02 (2.95,3.25)	3.05 (2.94,3.31)
OA	ICC (95% CI)	0.37 (0.22,0.50)	0.34 (0.05,0.57)	0.29 (-0.03,0.54)	0.47 (0.23,0.65)
	Median (95% CI)	3.61 (3.55,3.72)	3.71 (3.59,3.89)	3.64 (3.54,3.84)	3.43 (3.32,3.63)
**DIST**					
YA	ICC (95% CI)	0.83 (0.79,0.87)	0.85 (0.79,0.90)	0.82 (0.75,0.89)	0.83 (0.75,0.89)
	Median (95% CI)	3.93 (3.91,4.17)	3.86 (3.76,4.26)	3.83 (3.74,4.20)	4.07 (3.91,4.37)
OA	ICC (95% CI)	0.46 (0.33,0.58)	0.58 (0.39,0.73)	0.48 (0.25,0.66)	0.39 (0.13,0.61)
	Median (95% CI)	4.40 (4.27,4.43)	4.53 (4.27,4.57)	4.33 (4.16,4.46)	4.37 (4.18,4.44)
**COM**					
YA	ICC (95% CI)	0.82 (0.78,0.86)	0.71 (0.59,0.81)	0.87 (0.81,0.91)	0.85 (0.79,0.90)
	Median (95% CI)	4.00 (3.86,4.08)	4.00 (3.82,4.14)	4.04 (3.77,4.20)	3.96 (3.73,4.16)
OA	ICC (95% CI)	0.77 (0.72,0.82)	0.65 (0.49,0.77)	0.77 (0.66,0.85)	0.75 (0.64,0.84)
	Median (95% CI)	4.21 (4.08,4.29)	4.64 (4.40,4.68)	4.21 (4.05,4.40)	3.75 (3.63,3.95)
**DOM**					
YA	ICC (95% CI)	0.75 (0.69,0.80)	0.67 (0.52,0.78)	0.76 (0.77,0.85)	0.81 (0.73,0.88)
	Median (95% CI)	3.86 (3.80,4.01)	3.79 (3.68,4.01)	3.90 (3.75,4.11)	3.88 (3.74,4.16)
OA	ICC (95% CI)	0.48 (0.35,0.59)	0.54 (0.34,0.70)	0.35 (0.06,0.58)	0.40 (0.14,0.61)
	Median (95% CI)	4.29 (4.18,4.34)	4.18 (4.07,4.36)	4.50 (4.37,4.62)	4.12 (3.93,4.20)
**TRUST**					
YA	ICC (95% CI)	0.81 (0.77,0.85)	0.70 (0.57,0.80)	0.87 (0.81,0.92)	0.82 (0.74,0.88)
	Median (95% CI)	3.82 (3.76,3.98)	4.07 (3.90,4.19)	3.73 (3.56,3.98)	3.64 (3.60,3.98)
OA	ICC (95% CI)	0.69 (0.62,0.76)	0.57 (0.39,0.72)	0.61 (0.44,0.75)	0.75 (0.64,0.84)
	Median (95% CI)	4.20 (4.09,4.26)	4.53 (4.40,4.63)	4.03 (3.97,4.24)	3.87 (3.75,4.07)

*ICC, intra-class correlation coefficient using two-way random model with measures of consistency, 95% CI = 95% confidence interval; ATT, attractiveness rating dimension; FAM, familiarity rating dimension; DIST, distinctiveness rating dimension; COM, competence rating dimension; DOM, dominance rating dimension, TRUST, trustworthiness rating dimension; YA, younger adults; OA, older adults; Y, younger faces; M, middle-age faces; O, older faces.*

**FIGURE 1:**
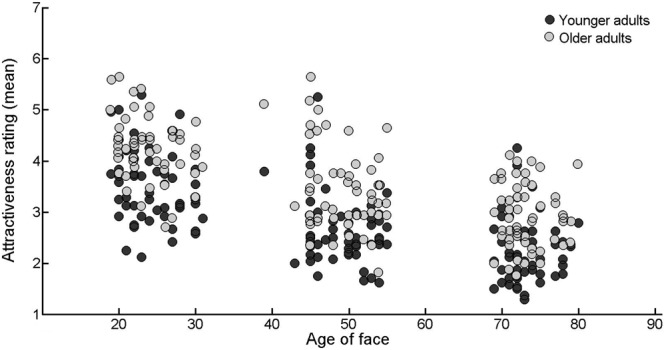
**Scatterplot showing the correlation between mean attractiveness ratings and (actual) age of face**.

First, we wished to determine whether the attractiveness ratings of younger and older adults were associated to each other, and to the actual and perceived age of the faces. The actual and perceived age of the face were taken from the FACES face database (Ebner et al., 2010). The perceived age of the face was based on the average of the age estimates collected from younger and older adults. We found that the attractiveness ratings provided by the younger and older participants’ were positively correlated (*r* = 0.82, *p* < 0.001). As shown in **Figure 1**, attractiveness ratings provided by both the younger and older adults decreased with the increasing age of the face (*r* = -0.62, *p* < 0.001 and *r* = -0.64, *p* < 0.001, respectively). Consistent with this result, a negative correlation was also found between the participants’ perceived age of the face and the attractiveness ratings provided by the younger and older adults (*r* = -0.65, *p* < 0.001 and *r* = -0.67, *p* < 0.001, respectively).

Next we conducted a multilevel random coefficient model on the attractiveness ratings, as it accounts for the hierarchical structure of the data (i.e., ratings of faces nested within participants). The linear regression model was utilized in conjunction with a restricted maximum likelihood estimation method to fit the expected model to the data. The main components of these models included the fixed part (i.e., the average effects of the age of the participant, average effects of the age of the face, and a cross-level interaction between the age of the participant by age of the face) and the random effects (i.e., variance components; age of face and residual).

The results of the multilevel random coefficient model are summarized in **Table 3**. They revealed that older participants rated the faces as significantly more attractive compared to younger participants (*γ* = 0.80, *p* < 0.05). The size of the effect can be interpreted by comparing the size of the regression coefficient with the standard deviation of the mean rating for attractiveness across all faces and all participants (*SD* = 0.68). This comparison showed that the difference between the ratings by the two age groups was over one standard deviation. Furthermore, the attractiveness ratings given by both age groups decreased with increasing age of the face (*γ* = -0.60, *p* < 0.05), with the size of the effect being almost one standard deviation.

**Table 3:** Multilevel random coefficient model on the attractiveness ratings and on the perceptual and social attribute ratings.

	ATT	FAM	DIST	COM	DOM	TRUST
Fixed effects						
Intercept	3.25ˆ*	3.26ˆ*	3.37ˆ*	3.08ˆ*	3.16ˆ*	3.47ˆ*
Age of participant	0.80ˆ*	0.32	0.56ˆ*	0.92ˆ*	0.62ˆ*	0.66ˆ*
Age of face	-0.60ˆ*	-0.21	0.18	0.34ˆ*	0.20	0.05
Age of participant x age of face	-0.03	0.04	-0.12	-0.35ˆ*	-0.14	-0.18
Random effects						
Intercept	0.50ˆ*	0.81ˆ*	0.30	0.69ˆ*	0.27ˆ*	0.35ˆ*
Covariance between intercept and Age of face	-0.13	-0.07	-0.07	-0.11	-0.09	-0.12
Age of face	0.09ˆ*	0.03	0.05	0.04ˆ*	0.07ˆ*	0.08ˆ*
Residual	0.11ˆ*	0.10ˆ*	0.12ˆ*	0.05ˆ*	0.09ˆ*	0.11ˆ*
AIC	228.70	180.01	180.48	160.41	178.43	182.52

*ATT, attractiveness rating dimension; FAM, familiarity rating dimension; DIST, distinctiveness rating dimension; COM, competence rating dimension; DOM, dominance rating dimension; TRUST, trustworthiness rating dimension; AIC, Akaike Information Criteria. Young participants coded as 1, older participants as 2. Young faces coded as 1, middle-aged as 2, old as 3. ^∗^denotes *p* < 0.05.*

### Perceptual Attributes Determining the Attractiveness Ratings of the Older and Younger Adults to the Young, Middle-Aged and Older Faces

An inter-rater reliability score (i.e., ICC) was calculated on the ratings provided for the distinctiveness and familiarity dimensions, which are displayed in **Table 2**. Younger participants showed a high inter-rater reliability score for the distinctiveness dimension (*α* > 0.80), but their familiarity ratings were less consistent (*α* = 0.69). Older adults had low inter-rater reliability scores for distinctiveness (*α* < 0.50) although, interestingly, the scores were highest to the younger than older faces (*α* = 0.58). Older adults’ inter-rater reliability scores were also low for the familiarity dimension (*α* < 0.40). Moreover, 95% confidence intervals indicated that older adults’ ratings were more variable overall compared to those of younger adults.

We then investigated whether the both the familiarity and distinctiveness ratings provided by the younger and older participants were related to each other, and to the actual and perceived age of the face. The ratings of younger and older participants were moderately correlated for both distinctiveness (*r* = 0.56, *p* < 0.001) and familiarity (*r* = 0.48, *p* < 0.001). There was a weak, negative correlation between the familiarity ratings of both younger and older adults and the actual age of face (*r* = -0.25, *p* < 0.01 and *r* = -0.23, *p* < 0.01, respectively). Consistently, a negative correlation was found between the perceived age of the face and the familiarity ratings of both the young and older participants (*r* = -0.28, *p* < 0.001 and *r* = -0.26, *p* = 0.001, respectively). The distinctiveness ratings did not correlate with either the actual or perceived age of face for either of the participant groups (*p* > 0.05).

**FIGURE 2:**
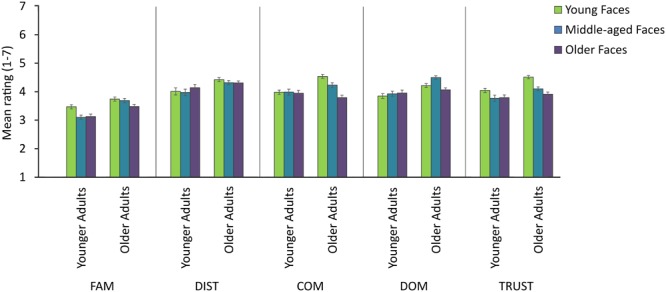
**Mean ratings (SE) of attractiveness and its perceptual and social attributes provided by the younger and older adults to the younger, middle-aged and older faces**. FAM, familiarity rating dimension; DIST, distinctiveness rating dimension; COM, competence rating dimension; DOM, dominance rating dimension; TRUST, trustworthiness rating dimension.

We performed separate, multilevel random coefficient models for the perceptual attribute dimensions of familiarity and distinctiveness (see **Table 3**; **Figure 2**). This analysis revealed that older adults provided higher distinctiveness ratings to the faces overall (*γ* = 0.56, *p* < 0.05). The difference between the ratings of the two participant age groups was approximately three quarters of one standard deviation (*SD* = 0.70). There were no significant effects of age on the ratings to the familiarity dimension.

In order to examine whether age and the perceptual qualities of the faces had an effect on attractiveness ratings, we conducted separate multilevel random coefficient models (see **Table 4**). We categorized the face images into familiarity and distinctiveness groups of faces (i.e., least familiar/distinctive faces coded as 1, most familiar/distinctive faces as 2) by performing a median split analysis on either the familiarity or distinctiveness ratings of each sex and age group of the face images. We chose to perform the median split analysis on each sex and age group of the faces separately in order to control for possible age and sex effects on the results. A median split approach was chosen because the participants provided ratings for two dimensions only, i.e., not all participants rated faces for attractiveness. Each model included the fixed part (i.e., the average effects of the age of the participant, average effects of the age of the face, average effect of the perceptual quality group of the face and all the two-way and a three-way interactions between these three variables) and the random effects (i.e., variance components; age of face, perceptual quality group of face, covariance estimates and residual). Each participant’s trait rating was the dependent variable.

**Table 4:** Multilevel random coefficient models on the attractiveness ratings with age of participant, perceptual group of face and age of face as factors.

	FAM	DIST
Fixed effects		
Intercept	1.98ˆ*	3.14ˆ*
Age of participant	1.17ˆ*	0.62
PERC group of face	0.86ˆ*	0.05
Age of face	-0.46	-0.54
Age of participant × PERC group of face	-0.25	0.14
Age of participant × Age of face	-0.25	0.21
PERC group of face × Age of face	-0.10	-0.03
Age of participant × PERC group of face × Age of face x	0.15	-0.16
Random effects		
Intercept	0.56ˆ*	0.63ˆ*
Covariance between intercept and PERC group of face	-0.05	-0.04
PERC group of face	0.02	0.01
Covariance between intercept and Age of face	-0.18ˆ*	-0.14ˆ*
Covariance between PERC group of face and Age of face	0.04ˆ*	-0.01
Age of face	0.10ˆ*	0.11ˆ*
Residual	0.11ˆ*	0.11ˆ*
AIC	394.42	375.79

*PERC, perceptual quality; FAM, effects for familiarity rating dimension; DIST, effects for distinctiveness rating dimension; AIC, Akaike Information Criteria. Young participants coded as 1, older participants as 2. Young faces coded as 1, middle-aged as 2, old as 3. Least familiar faces coded as 1, most familiar as 2. Least distinctive faces coded as 1, most distinctive as 2. ^∗^denotes *p* < 0.05.*

The multilevel random coefficient model including familiarity face groups revealed that most familiar faces received higher attractiveness ratings (*γ* = 0.86, *p* < 0.05). Furthermore, and consistent with our earlier analyses, older adults gave higher attractiveness ratings overall relative to younger adults (*γ* = 1.17, *p* < 0.05). For the model including the distinctiveness face groups, there were no statistically significant effects, however, there was a near significant effect that older faces were rated as less attractive than younger faces (*γ* = -0.54, *p* = 0.06).

### Social Attributes Determining the Attractiveness Ratings of the Older and Younger Adults to the Young, Middle-Aged and Older Faces

An inter-rater reliability score (i.e., ICC) was calculated from the ratings provided for the competence, dominance and trustworthiness dimensions and ICC coefficients are displayed in **Table 2**. The inter-rater reliability was high for younger participants to the dimensions of competence and trustworthiness (*α* > 0.75), however, their ratings were less consistent for the dominance dimension related to the young face stimuli (*α* = 0.67). There was a high inter-rater reliability for the older adults ratings of competence (*α* = 0.77) and relatively high inter-rater reliability for ratings of trustworthiness (*α* = 0.69). However, older adults’ inter-rater reliability scores were low for the dominance dimension (*α* = 0.48). Older adults’ ratings were more variable overall as indicated by the 95% confidence intervals shown in **Figure 2**.

We then examined whether the ratings along the social attributes provided by the younger and older adults were associated to each other, and whether the ages of the face stimuli affected the association between ratings of attractiveness and the ratings to other social attribute dimensions of competence, dominance and trustworthiness. We found moderate correlations between the ratings provided by the younger and older participants on the competence (*r* = 0.59, *p* < 0.001), dominance (*r* = 0.54, *p* < 0.001) and trustworthiness (*r* = 0.65, *p* < 0.001) dimensions. Furthermore, the ratings of competence provided by older adults were moderately and negatively correlated with the actual and perceived age of face (*r* = -0.48, *p* < 0.001 and *r* = -0.49, *p* < 0.001, respectively). However, the competence ratings of younger adults were not correlated with the age of the face (*p* > 0.05). There was a moderately negative correlation between older participants’ trustworthiness ratings and the actual (*r* = -0.45, *p* < 0.001) and perceived (*r* = -0.48, *p* < 0.001) age of face. The trustworthiness ratings of younger participants were weakly but negatively correlated with actual (*r* = -0.16, *p* < 0.05) and perceived (*r* = -0.21, *p* < 0.01) age of face. The dominance ratings did not correlate with age of face (*p* > 0.05).

Next we conducted separate multilevel random coefficient models for each of the social attribute dimensions of competence, dominance and trustworthiness to investigate the role of age on these trait dimensions (see **Table 3**; **Figure 2**). For competence, the multilevel random coefficient model revealed that, overall, older adults provided higher ratings relative to younger adults (*γ* = 0.92, *p* < 0.05). Older adults’ competence ratings were over one standard deviation higher compared to younger adults’ ratings (*SD* = 0.65). Older adults rated competence to decrease with increasing age of face (*γ* = -0.35, *p* < 0.05) and this difference amounted to approximately half of a standard deviation. For the dominance and trustworthiness dimensions overall, older adults provided higher ratings than younger adults (*γ* = 0.62, *p* < 0.05 and *γ* = 0.66, *p* < 0.05, respectively), and the difference between the participant groups was over one standard deviation in both dimensions (*SD* = 0.58 and *SD* = 0.59, respectively).

To examine the role of both age and attractiveness further for each social attribute (competence, dominance, trustworthiness) we conducted separate, multilevel random coefficient models (see **Table 5**). Specifically, we categorized the face images into separate attractiveness groups of faces (least attractive faces coded as 1, most attractive faces as 2) by performing a median split analysis on the attractiveness ratings of each sex and age group of the face images. Each model included the fixed part (i.e., the average effects of the age of the participant, average effects of the age of the face, average effect of the attractiveness group of the face and all the two-way and a three-way interactions between these three variables) and the random effects (i.e., variance components; age of face, attractiveness group of face, covariance estimates and residual). Each participant’s trait rating served as the dependent variable.

**Table 5:** Multilevel random coefficient models on the cognitive trait dimension ratings with age of participant, attractiveness group of face and age of face and as factors.

	COM	DOM	*TRUST*
Fixed effects			
Intercept	2.99ˆ*	4.18ˆ*	2.54ˆ*
Age of participant	0.88	0.38	0.86ˆ*
ATT group of face	0.05	-0.65	0.62
Age of face	-0.29	-0.17	-0.24
Age of participant × ATT group of face	0.03	0.14	-0.14
Age of participant × Age of face	-0.24	-0.11	-0.10
ATT group of face × Age of face	0.43ˆ*	0.22	0.19
Age of participant × Age of face × ATT group of face	-0.08	-0.01	-0.05
Random effects			
Intercept	0.70ˆ*	0.15	0.27ˆ*
Covariance between intercept and ATT group of face	-0.05	0.02	0.01
ATT group of face	0.06ˆ*	0.02	0.01
Covariance between intercept and Age of face	-0.07	-0.05	-0.10ˆ*
Covariance between ATT group of face and Age of face	-0.03	-0.02	-0.02
Age of face	0.05ˆ*	0.07ˆ*	0.08ˆ*
Residual	0.10ˆ*	0.18ˆ*	0.11ˆ*
AIC	336.26	389.63	354.80

*COM, effects for competence rating dimension; DOM, effects for dominance rating dimension; TRUST, effects for trustworthiness rating dimension; AIC, Akaike Information Criteria. Young participants coded as 1, older participants as 2. Young faces coded as 1, middle-aged as 2, old as 3. Least attractive faces coded as 1, most attractive as 2. ^∗^denotes *p* < 0.05.*

For competence, the multilevel random coefficient model including attractiveness face groups revealed that an interaction between the age of the face and attractiveness group of the face increased the competence ratings (*γ* = 0.43, *p* < 0.05), indicating that the combined effect of attractiveness and the age of the face influenced perceived competence. Older adults provided higher trustworthiness ratings than younger adults (*γ* = 0.86, *p* < 0.05), a similar positivity effect was also apparent in competence ratings, however, it did not reach statistical significance (*γ* = 0.88, *p* = 0.06). The difference between the participant groups was over one standard deviation in trustworthiness (*SD* = 0.59). There were no significant effects for dominance dimension.

### Probability of Differentiation Underlying the Differences between Rating Provided by Older and Younger Adults to the Young, Middle-Aged and Older Faces

One approach to investigate further the processes related to the positivity bias of older adults, is to examine whether this effect is mediated by a decline in cognitive processing in older adulthood. As such, we calculated the probability of differentiation (*P*_D_) scores as described in the Methods section (more details in Linville et al., 1986; Ng et al., 2014). These scores reflect how different the participants perceive the faces to be from one another, that is, a higher probability of differentiation score means that the faces are perceived as more different from one another.

**FIGURE 3:**
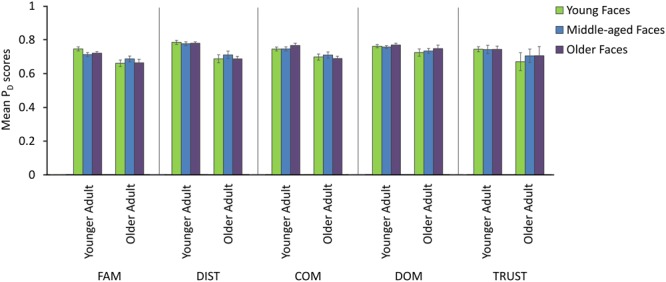
**Mean probability of differentiation scores (SE) of younger and older adults to the young, middle-aged and older faces.** FAM, familiarity rating dimension; DIST, distinctiveness rating dimension; COM, competence rating dimension; DOM, Dominance rating dimension; TRUST, trustworthiness rating dimension.

We conducted separate analyses, for each of the rating dimensions, to examine the effects of the age of the participant and the age of the face had on the differentiation performance. This was achieved by a mixed-factor ANOVA in which the age of the participant was a between-subjects factor, the age of the face a within-subjects factor and the ratings provided to each dimension as the dependent variable. The findings are displayed in **Figure 3**.

For the attractiveness dimension, the participants differentiated the attractiveness of younger (*M* = 0.73) and middle-aged (*M* = 0.70) faces more than that of older faces [*F*_1.6,60.9_= 11.64, *MSE* = 0.00, *p* < 0.001, η_p_^2^ = 0.23; Greenhouse–Geisser corrected; *χ*^2^(2) = 12.48, *p* < 0.01, *𝜀* = 0.78; Bonferroni *post hoc* tests *p* < 0.05]. There was a near significant interaction between the age of the face and the participant [*F*_1.6,60.9_= 2.95, *MSE* = 0.00, *p* = 0.052, η_p_^2^ = 0.08; Greenhouse–Geisser corrected; *χ*^2^(2) = 12.48, *p* < 0.01, *𝜀* = 0.78; Bonferroni *post hoc* tests *p* < .05]. This interaction suggested that younger adults had decreasing levels of differentiation with an increasing age of the face, whereas older adults had roughly similar levels of differentiation for all the ages of the faces. There was no main effect of the age of the participant (*F*_1,39_= 2.36, *MSE* = 0.00, *p* > 0.01, η_p_^2^ = 0.06).

Both the age of the participant and age of the face influenced the probability of differentiation scores for the social attributes of competence and trustworthiness. Specifically, older adults showed significantly less differentiation for competence (*M* = 0.71) relative to younger adults (*M* = 0.77) (*F*_1,35_ = 7.86, *MSE* = 0.01, *p* < 0.01, η_p_^2^ = 0.18; Bonferroni *post hoc* tests *p* < 0.01). In addition, an interaction effect between the age of the face and participant (*F*_2,70_= 5.15, *MSE* = 0.00, *p* < 0.01, η_p_^2^ = 0.13) revealed that younger adults’ differentiation scores of older faces (*M* = 0.78) were higher than those of older adults for younger and older adult faces (*M* = 0.71 and 0.70, respectively; Bonferroni corrected *post hoc* tests, *p* < 0.05). There was no main effect of the age of the face for the scores on the competence dimension (*F*_2,70_ = 0.55, *MSE* = 0.00, *p* > 0.05, η_p_^2^ = 0.08).

For the trustworthiness dimension, older adults showed significantly less differentiation overall (*M* = 0.71) in comparison to younger adults (*M* = 0.76) (*F*_1,35_ = 6.99, *MSE* = 0.01, *p* < .05, η_p_^2^ = 0.17; Bonferroni *post hoc* tests *p* < 0.05). The main effect of age of face [*F*_1.7,58.3_ = 4.27, *MSE* = 0.00, *p* < 0.05, η_p_^2^ = 0.11; Greenhouse–Geisser corrected; *χ*^2^(2) = 7.58, *p* < .05, *𝜀* = 0.83] was driven by an interaction between age of face and age of participant [*F*_1.7,58.3_ = 4.86, *MSE* = 0.00, *p* < 0.05, η_p_^2^ = 0.12; Greenhouse-Geisser corrected; *χ*^2^(2) = 7.58, *p* < 0.05, *𝜀* = 0.83]. Bonferroni-corrected *post hoc* tests revealed that older adults were less able to differentiate the trustworthiness of younger faces (*M* = 0.68) compared to middle-aged and older faces (*M* = 0.72 and 0.72, respectively, *p* < 0.05), whereas younger adults’ differentiation scores were the same for all faces (all *Means* = 0.76). There were no significant effects for dominance ratings.

The analyses with the perceptual attributes revealed a significant interaction between age of face and age of participant for the familiarity dimension [*F*_1.6,50.3_ = 3.54, *MSE* = 0.002, *p* < 0.05, η_p_^2^ = 0.10; Greenhouse-Geisser corrected; *χ*^2^(2) = 7.95, *p* < 0.05, *𝜀* = 0.81]. Although Bonferroni-corrected *post hoc* tests were not significant, it seemed that the differentiation scores of younger adults were higher in comparison to those of older adults for younger faces (*M* = 0.76 vs. 0.67, respectively). For the distinctiveness dimension, older adults showed significantly less differentiation for all face ages (*M* = 0.71) compared to younger adults (*M* = 0.79) (*F*_1,34_ = 25.54, *MSE* = 0.01, *p* < .001, η_p_^2^ = 0.43; Bonferroni *post hoc* tests *p* < 0.001). There were no other significant effects for the familiarity and distinctiveness dimensions (*p* > 0.05).

## Discussion

### Aesthetic Preferences for Faces Are Determined by Cues Signaling Youthfulness

The main aim of the present study was to elucidate how preferences for faces change with aging and whether there is evidence for a bias in favoring the faces matching one’s own age group. We also investigated whether the perceptual attributes of faces (distinctiveness and familiarity) influenced the role of age on perceived facial attractiveness, as these attributes have not previously been reported in other studies. We were also interested in whether aesthetic preferences, together with age, influence our evaluations of the social attributes determined from faces and how age and the perceptual qualities of face, familiarity and distinctiveness, affect the perceived attractiveness of a face. To that end, we used a robust data-driven approach, and included the most central social attributes found in previous research on the evaluation of faces (Oosterhof and Todorov, 2008; Sutherland et al., 2013; Todorov et al., 2015).

Our findings revealed a clear preference for youthful faces by all age groups. Specifically we found that attractiveness ratings decreased with the increasing age of faces. This finding is consistent with that previously reported by Ebner (2008). Interestingly, older adults’ ratings of perceived competence and trustworthiness also reduced with an increasing age of the faces. Furthermore, older participants’ ratings were higher overall compared to those provided by younger adults. This finding is consistent with the previously reported ‘positivity bias’ in older adults (Mather and Carstensen, 2005; Ebner, 2008; Zebrowitz et al., 2013). However, in contrast to what we expected based on previous reports, we failed to find support for the same-age bias on aesthetic preferences (Wright and Stroud, 2002; Anastasi and Rhodes, 2005; Rhodes and Anastasi, 2012). Instead, both the younger and older observers preferred youthful faces and all participants showed a high agreement in their responses. Our results therefore suggest that aesthetic preferences in person perception are mainly driven by the (perceived or actual) age of a face and not by a bias toward the observer’s own reference-group.

One of the reasons why youthful faces are preferred may be that these faces have fewer age-specific features (e.g., wrinkles, blemishes) that may decrease the clarity of cues signaling socially relevant information. Thus, information from youthful-looking faces may be faster to process, which, according to the perceptual fluency theory (see, for example, Reber et al., 2004) can be thought to increase our preferences for such faces regardless of the age of the perceiver. The ease of processing other perceptual qualities, such as the averageness or symmetry of a face (e.g., Pisanski and Feinberg, 2013), may also explain the preference for younger-looking faces. Faces of younger adults can be thought to typically contain cues that are more average and symmetrical compared to older faces. On the other hand, symmetry and averageness are also features indicating the reproductive value of a face, as they suggest good health, fitness and a strong immune system (Thornhill and Gangestad, 1999; Lie et al., 2008; Pisanski and Feinberg, 2013). However, the extent to which perceived health predicts aesthetic evaluations from faces is unclear, with some studies suggesting it plays little or no predictive role on judgements of facial attractiveness (Kalick et al., 1998; Rhodes et al., 2003). Instead, our data suggest that face age is a strong determinant of facial attractiveness, with preferences for younger faces across all ages of observers. Although youthfulness, in particular, is a strong cue for determining reproductive health, it was previously unclear whether this attribute may have an important influence on judgments of facial attractiveness across different age groups. However, it is also unclear why the age of the observer does not affect the preference for age of faces, since participants of all age groups preferred younger faces. Future studies are needed to investigate the specific role of youthfulness on attractiveness and whether or not other perceptual or social attributes may be driving preferences for these faces.

### Aesthetic Preferences Are Associated with Perceived Social Attributes of Faces

The second objective of the present study was to investigate the perceptual and social attributes of aesthetic preferences in person perception and whether they would show age-related differences. Again, the ratings of the younger participants had high inter-rater agreement, whereas older participants had lower inter-rater consistency in their ratings and were more variable in their responses than younger adults. Interestingly, the perceptual attributes of aesthetic preferences did not suggest changes dependent on the age of face. Older adults provided higher ratings on perceptual dimensions compared to younger adults. Our findings from the multilevel random coefficient models on the attractiveness ratings, including the familiarity group of the faces as one of the factors, are in line with the well-known ‘mere exposure’ effect in which the increasing familiarity of a face is associated with higher levels of attractiveness (e.g., Peskin and Newell, 2004). Moreover, the correlational analyses showed that both younger and older participants rated older faces to be less familiar, however, this association was weak. We found no evidence for combined effects of age and familiarity on aesthetic preferences. Similarly, distinctiveness ratings, together with age-related factors, did not affect the perceived attractiveness of the faces.

Interestingly, our results suggest that a combined effect of older age and higher attractiveness leads to positive evaluations of competence. Previous research indicated similar positive associations between attractiveness and competence (Langlois et al., 2000). This highlights the importance of taking into account the links between aesthetic preferences and age-related factors, as the correlational analysis focusing only on age showed the ratings of competence to reduce with increasing age of the face. This reduction in the perceived competence of older faces may be related to attitudes and stereotypes of older adults (e.g., see Kite and Johnson, 1988; Kite et al., 2005; and Fiske et al., 2007 for a review), however, it appears that possessing a highly attractive face in older age might actually lead to positive competence evaluations, potentially focusing on more advantageous aspects of aging, such as life experience and wisdom. On the other hand, these results may be based on perceptual effects with evidence suggesting that sensitivity to facial cues varies depending on the trait and age of the face being evaluated. For example, health cues have been suggested to be more apparent in older faces and hostility cues in younger faces (Zebrowitz et al., 2013). However, factors such as stereotypes and (cultural) expectations are likely to interact with these outer, physical facial features. For example, some studies have shown that ‘halo effects’ are stronger for traits that are more culturally valued, such as competence and health (Wheeler and Kim, 1997; Shaffer et al., 2000; Zebrowitz and Franklin, 2014).

The ratings provided to the dominance and trustworthiness dimensions were not affected by the attractiveness ‘halo’ effect. Instead, the ratings for dominance and trustworthiness, shown to be two of the most central dimensions for the perception of traits from faces besides the attractiveness-health dimension (Oosterhof and Todorov, 2008; Sutherland et al., 2013; Todorov et al., 2015), were not affected by the age of the faces according to the results of the models.

According to the ecological theory (see a review by Zebrowitz and Montepare, 2008) the high inter-rater agreement in the ratings of the social attributes indicates that cues in the structural appearance of a face signal consistent social information to others. This ability may stem from our need to perceive ecologically relevant social information from faces, including health and age (Mcarthur and Baron, 1983; Zebrowitz and Collins, 1997; Zebrowitz and Montepare, 2008). In other words, neutral-expressive faces can be perceived to contain physical features that cause them to, for example, resemble a specific health status or age group (Zebrowitz and Montepare, 2008). For instance, these ‘overgeneralization’ processes can cause people to misattribute similarity in facial cues related to health (e.g., pale skin color and uneven skin texture signaling sickness) and age (e.g., wrinkles signaling older age) to personality traits (Zebrowitz and Montepare, 2008). Zebrowitz and Montepare (2008) also suggested perceptions made from older faces can be partially influenced by the anomalous face overgeneralization effect as older faces may be structurally similar to anomalous (e.g., unhealthy) faces, which may, in turn, contribute to the negative inferences made about their character traits. Furthermore, some studies investigating social impressions made from voices (Montepare et al., 2014) have suggested that there may be a supramodal ‘elderly overgeneralization effect’ in that younger perceivers may respond to older-sounding, -looking and –moving younger adults similarly as they would to an older person. Eventually these first impressions are then thought to serve as adaptive signals to make important decisions about whether to approach or avoid another person, an ability that is crucial for survival (Zebrowitz and Montepare, 2008). This has led some researchers to propose that the attractiveness ‘halo’ effect is more driven by the perception that ‘ugly is bad,’ and less by the ‘beautiful is good’ principle (Griffin and Langlois, 2006).

### Differences between Older and Younger Adults’ Aesthetic Preferences and Other Social Attributions of Faces

With regards to age of the participants, we found no evidence for an own-age bias contrary to previous reports. However, consistent with previous findings, older adults exhibited a positivity bias in their ratings to the different social attributes of unfamiliar faces, particularly competence and trustworthiness, (e.g., Ebner, 2008; Zebrowitz et al., 2013). Although this positivity bias may be due to several reasons, we decided to first examine it in more detail using an approach reported in a study by Ng et al. (2014), known as the probability of differentiation. This approach is proposed as an estimate of the neural differentiation of the representations of faces that is thought to be less distinct in older adulthood (Baltes et al., 1980; Salthouse et al., 1996). Our findings revealed the consistent pattern that older adults showed less differentiation in their ratings than younger adults (except for ratings to the dominance dimension). Ng et al. (2014) also reported that older adults perceived faces as less different from one another (for impressions of health, hostility, untrustworthiness, and competence). This lack of making fine distinctions between face stimuli may then be reflected in older adults’ more positive impressions, possibly leading to the more restricted use of the rating scale, which is subsequently seen in their higher ratings. Alternatively, it might also be the case that having more experience over one’s life with a rich variety of faces may lead older adults to weigh the perceptual dimensions of a face less when assessing its social characteristics. In that case, although differences between faces may be perceived by older adults, these percepts may not necessarily drive the differences in the ratings of some social traits. However, other research suggests that a decline in perceptual differentiation may lead to changes in the ability to evaluate other socially relevant information from faces. For example, the relatively poor recognition of facial expressions in older adults may arise from the age-related changes affecting perceptual processes and processing demands on associated brain mechanisms (Ruffman et al., 2008; Franklin and Zebrowitz, 2013). Our results are therefore consistent with this neural differentiation approach and suggest that low differentiation at a perceptual level may also affect the social evaluation of faces by older adults.

A further possibility for the more positive ratings provided by older adults, as argued by other researchers, is that the tendency for older adults to use relatively more positive ratings may be explained by age-related changes in motivation. Specifically, it has been suggested that older adults may be more motivated to maintain a positive mood because of their relatively short future perspective and thus may be less responsive to negative information (e.g., Carstensen, 1995; Carstensen et al., 1999; Carstensen and Mikels, 2005; Mather and Carstensen, 2005; Ruffman et al., 2006; Kellough and Knight, 2012).

Another interesting difference between older and younger adults in the present study was that older adults were less consistent in their ratings of dominance and trustworthiness than for other attributes of the faces. This reduced consistency in the ratings from the older adult cohort may be due to the more substantive life experience of older adults, which makes them less willing to form impressions of other people’s traits and qualities based on just appearance (e.g., Baltes and Smith, 2008). This is a good strategy as the evidence for the accuracy of first impressions is mixed (Todorov et al., 2015). It may also be suggested that having more life experience may cause older adults to view the sample of faces used in this study as overall less distinctive with respect to the features of all possible faces. However, due to the lack of consistency of older adults also in their ratings of the perceptual qualities of the faces (distinctiveness and familiarity) it may be suggested that the lower inter-rater scores of older adults may be linked to either the lack of differentiation between the representations of the face stimuli (Lee et al., 2014) or to the internal representations of each of the faces on preference-relevant dimensions to be noisy or overlapping. Both of these effects could possibly lead to changes in judgments of repeated stimuli resulting in low inter-rater correlations.

## Conclusion

With the increasing size of the older adult population in Western societies, and the social impact of perceiving traits in unfamiliar faces, a better understanding of the age-related factors on face perception is of great importance. In our study we found that the age of the face, as well as the age of the perceiver, affects the formation of aesthetic preferences for, and social evaluations of, unfamiliar faces. Specifically, our results suggest a consistent aesthetic preference for youthful faces across all age groups. Older adults also provided higher ratings of perceived competence and trustworthiness with a decreasing age of the faces. As such, for the first time, our results also provide some evidence for an interaction between the influence of attractiveness and the perceived age of a face on competence judgments. This highlights the importance of considering aesthetic preferences and age factors together, and not in isolation, when examining the formation of first impressions. We replicated previous findings that familiar faces were perceived as more attractive; however, this effect was not modulated by the age of the face.

Although we found no evidence that participants had a bias in favoring faces belonging to their own age group, nevertheless, the age of the participant was important for eliciting preferences and in determining their social and perceptual attributes. Older adults provided higher ratings overall relative to younger adults, most likely due to age-related changes in cognitive processing or motivation. These preferences, as well as the perceptual and social attributions perceived in unfamiliar faces, may have consequences in our everyday lives (Cornwell et al., 2006; Olivola et al., 2014). For example, there is some evidence to suggest that attractive people are given preferential treatment in many areas of life, including personal relationships, and work life (Langlois et al., 2000). Therefore, together with existing evidence (e.g., Ebner, 2008; Zebrowitz et al., 2013) our findings stress the importance of age on preferences for faces and have implications for understanding everyday social decision making based on information perceived from faces.

## Author Contributions

FN, HK, and BC participated in the conception and design of the study. HK, BC, and SC participated in the acquisition and analysis of the data. HK, FN, and BC participated in the interpretation of data. HK and BC wrote the first draft of the manuscript, and FN, HK, and BC revised the manuscript critically for important intellectual content and prepared it for submission. HK, BC, SC, and FN gave final approval of the manuscript to be submitted to the journal Frontiers in Human Neuroscience. All authors agree to be accountable for all aspects of the work in ensuring that questions related to the accuracy or integrity of any part of the work are appropriately investigated and resolved.

## Conflict of Interest Statement

The authors declare that the research was conducted in the absence of any commercial or financial relationships that could be construed as a potential conflict of interest.
